# Associations between polygenic risk scores for four psychiatric illnesses and brain structure using multivariate pattern recognition

**DOI:** 10.1016/j.nicl.2018.10.008

**Published:** 2018-10-09

**Authors:** Siri Ranlund, Maria Joao Rosa, Simone de Jong, James H. Cole, Marinos Kyriakopoulos, Cynthia H.Y. Fu, Mitul A. Mehta, Danai Dima

**Affiliations:** a Department of Neuroimaging, Institute of Psychiatry, Psychology and Neuroscience, King's College London, London, UK; b Department of Computer Science, University College London, London, UK; c NIHR BRC for Mental Health, Institute of Psychiatry, Psychology and Neuroscience, King's College London and SLaM NHS Trust, London, UK; d MRC Social, Genetic & Developmental Psychiatry Centre, Institute of Psychiatry, Psychology and Neuroscience, King's College London, London, UK; e Computational, Cognitive & Clinical Neuroimaging Laboratory, Division of Brain Sciences, Department of Medicine, Imperial College London, London, UK; f National and Specialist Acorn Lodge Inpatient Children Unit, South London and Maudsley NHS Foundation Trust, London, UK; g Department of Child and Adolescent Psychiatry, Institute of Psychiatry, Psychology and Neuroscience, King's College London, London, UK; h School of Psychology, University of East London, London, UK; i Centre for Affective Disorders, Institute of Psychiatry, Psychology and Neuroscience, King's College London, London, UK; j Department of Psychology, School of Arts and Social Sciences, City, University of London, London, UK

**Keywords:** ADHD, Autism, Bipolar disorder, Major depression, Schizophrenia, MRI

## Abstract

Psychiatric illnesses are complex and polygenic. They are associated with widespread alterations in the brain, which are partly influenced by genetic factors. There have been some attempts to relate polygenic risk scores (PRS) – a measure of the overall genetic risk an individual carries for a disorder – to brain structure using univariate methods. However, PRS are likely associated with distributed and covarying effects across the brain. We therefore used multivariate machine learning in this proof-of-principle study to investigate associations between brain structure and PRS for four psychiatric disorders; attention deficit-hyperactivity disorder (ADHD), autism, bipolar disorder and schizophrenia. The sample included 213 individuals comprising patients with depression (69), bipolar disorder (33), and healthy controls (111). The five psychiatric PRSs were calculated based on summary data from the Psychiatric Genomics Consortium. T1-weighted magnetic resonance images were obtained and voxel-based morphometry was implemented in SPM12. Multivariate relevance vector regression was implemented in the Pattern Recognition for Neuroimaging Toolbox (PRoNTo). Across the whole sample, a multivariate pattern of grey matter significantly predicted the PRS for autism (*r* = 0.20, p_FDR_ = 0.03; MSE = 4.20 × 10^−5^, p_FDR_ = 0.02). For the schizophrenia PRS, the MSE was significant (MSE = 1.30 × 10^−5^, p_FDR_ = 0.02) although the correlation was not (*r* = 0.15, p_FDR_ = 0.06). These results lend support to the hypothesis that polygenic liability for autism and schizophrenia is associated with widespread changes in grey matter concentrations. These associations were seen in individuals not affected by these disorders, indicating that this is not driven by the expression of the disease, but by the genetic risk captured by the PRSs.

## Highlights

• The polygenic risk score (PRS) for autism is predicted from grey matter brain volumes
• The PRS for schizophrenia is also predicted from grey matter brain structure
• A multivariate approach to detect patterns of grey matter is superior to univariate
• Brain volumes are not predictive of PRS for ADHD and bipolar disorder

## 1 Introduction

Common psychiatric illnesses have complex etiologies and are polygenic (Lee et al., 2013; Wray et al., 2014). Autism, bipolar disorder and schizophrenia consistently show high heritability estimates, both from twin studies (up to 90%) and molecular genetic studies (Geschwind and Flint, 2015; Sullivan et al., 2012). Attention-deficit hyperactivity disorder (ADHD) is however known to have relatively fewer genetic influences with environmental risk factors playing a greater role in disease liability, and heritability estimates for ADHD in adults ranges from 40% to 70% (Brikell et al., 2015; Burmeister et al., 2008; Franke et al., 2011).

Genetic variants associated with an increased risk of developing a range of psychiatric illnesses have started to be identified (Cross-Disorder Group of the Psychiatric Genomics Consortium, 2013; Marshall et al., 2016; Sklar et al., 2011; Stefansson et al., 2008; Stone et al., 2008); most notably for schizophrenia where over 100 common genetic variants have been significantly detected (Ripke et al., 2014). Progress has also been made for bipolar disorder, where a handful of common variants have been identified (Chen et al., 2013; Sklar et al., 2011). However, for other disorders such as ADHD and autism, very few common genetic risk variants have been reliably detected (Warrier et al., 2015). Furthermore, it is still largely unknown how genetic risk variants lead to the development of psychiatric symptoms; an important goal of psychiatric genetic research is therefore to clarify the mechanisms of these variants (Bogdan et al., 2017; Carter et al., 2017; Glahn et al., 2014; Hall and Smoller, 2010; Harrison, 2015).

The polygenic risk score (PRS) is an estimate of the combined effect of a large number of common genetic variants (single nucleotide polymorphisms, SNPs) associated with a trait, each with a very subtle individual effect (Chatterjee et al., 2016; Dima and Breen, 2015; Purcell et al., 2009; Wray et al., 2014). PRSs for psychiatric illnesses differ between patients and controls, thus providing a useful tool to measure genetic liability to psychiatric disorders (Bramon et al., 2014; Derks et al., 2012; Power et al., 2015; Purcell et al., 2009; Ranlund et al., 2017; Vassos et al., 2017), including non-affected individuals. Thus, associations with PRS are predicted to be independent of current disease state and can be seen in healthy participants who may not have developed a disorder.

We chose to investigate associations between PRSs for four major psychiatric disorders and brain structure. A wide range of both cortical and subcortical brain structural alterations have been observed in patients with psychiatric illnesses, including in ADHD (Frodl and Skokauskas, 2012; Hoogman et al., 2017; Nakao et al., 2011; Valera et al., 2007), autism (Cauda et al., 2011; Deramus and Kana, 2015; Duerden et al., 2012; Nickl-Jockschat et al., 2012), bipolar disorder (Arnone et al., 2009; Hibar et al., 2016; Selvaraj et al., 2012; Vita et al., 2009; Wise et al., 2016), and schizophrenia (Bora et al., 2011; Palaniyappan et al., 2015; Radua et al., 2012; Steen et al., 2006; van Erp et al., 2015). Furthermore, brain structure is influenced by genetic factors, with high heritability estimates for measures including grey matter volumes (Batouli et al., 2014; Glahn et al., 2007; Jansen et al., 2015; Peper et al., 2007), and studies have linked genetic risk for psychiatric disorders to variations in brain structure (Doyle et al., 2015; Lee et al., 2016; McIntosh et al., 2006; Turner et al., 2012; Van Haren et al., 2012).

Amongst the psychiatric illnesses, the schizophrenia PRS has been most widely investigated to date; and although some studies have found associations with brain structure (Neilson et al., 2016; Terwisscha van Scheltinga et al., 2013), others have found no significant associations (Papiol et al., 2014; Van der Auwera et al., 2015; Voineskos et al., 2016). Whalley and colleagues (2013) also investigated the PRS for bipolar disorder but found no significant associations with fractional anisotropy in white matter tracts. Lastly, Reus et al. (2017) found no associations between PRS for schizophrenia, bipolar disorder or depression in a range of brain structural measures including total grey matter, white matter and subcortical volumes.

These studies all used a univariate approach, where brain voxels or regions-of-interest are tested individually against the PRS. However, we know that the PRS is an estimate of the overall genetic risk an individual carries for a disorder. It is plausible to consider this risk acting throughout development and thus the influence on brain structure may best be captured by considering co-varied and distributed effects across the whole brain, rather than large, localized effects. Hence, a multivariate machine learning method – that considers the pattern of inter-relationships between measurements (e.g. grey matter volumes across the brain) – might be a more powerful method to detect subtle and spatially distributed patterns of alterations. The aim is not to localize effects in the brain or to identify specific regions that are predictive. PRSs were used recently in a multivariate machine learning setting to differentiate schizophrenia and/or bipolar disorder patients from healthy controls by combining the PRS with brain and cognitive variables (Doan et al., 2017). However, adding the PRS did not improve the classifying performance of the algorithm, implying that the PRS's predictive value is captured by brain variables (Doan et al., 2017).

In this study, we investigated the associations between brain structure and polygenic risk scores for four psychiatric disorders; ADHD, autism, bipolar disorder, and schizophrenia. We first present a univariate regression, followed by a multivariate analysis investigating whether the pattern of grey matter densities across the brain can predict an individual's PRS. We hypothesised that a multivariate approach would be more sensitive to detect associations between brain structure and PRSs compared to a univariate method, and that predictions would be stronger for highly heritable disorders with larger genetic influences like autism, schizophrenia and bipolar disorder, compared to ADHD that is known to be less heritable.

## 2 Methods and materials

### 2.1 Participants

The sample included 213 participants from two studies; 69 patients with major depressive disorder and 70 healthy controls from the BRCDECC study (Cole et al., 2012, Cole et al., 2013; Costafreda et al., 2013), and 33 patients with bipolar disorder and 41 healthy controls from the VIBES study (Dima et al., 2016a, b; Frangou et al., 2017). All participants were unrelated and of white European ancestry. Table 1 includes a summary of demographic and clinical information for the participants.

**Table 1:** Participants' characteristics.

	**Whole sample** N = 213	**Depression BRCDECC study** *N* = 139		**Bipolar VIBES study** *N* = 74	
		**Patients with depression** *N* = 69	**Healthy controls** *N* = 70	**Patients with bipolar disorder** *N* = 33	**Healthy controls** *N* = 41
**Age** (mean years ± SD; range)	45.91 ± 11.48 18–68	48.77 ± 8.29 29–68	51.13 ± 7.64 26–66	43.36 ± 11.34 21–63	44.22 ± 13.05 18–63
**Sex** (% female)	57.75%	71.01%	55.71%	48.48%	46.34%
**Full IQ** (WAIS-R)^a^	118.71 ± 13.37	117.34 ± 11.32	120.83 ± 8.91	118.23 ± 19.53	117.70 ± 17.13
**Age of onset**^b^ (years)	–	20.87 ± 9.28	–	23.79 ± 7.46	–
**Illness duration**^b^ (years)	–	30.59 ± 12.37	–	19.88 ± 10.66	–
**HDRS**	–	–	–	2.47 ± 2.83	0.25 ± 0.63
**YMRS**	–	–	–	1.07 ± 2.62	0.18 ± 0.50
**BDI**	–	15.16 ± 11.30	1.70 ± 1.63	–	–

Continuous data shown as mean ± standard deviations (SD). WAIS-R = Wechsler Adult Intelligence Scale – Revised version; HDRS = Hamilton Depression Rating Scale; YMRS = Young Mania Rating Scale; BDI = Beck Depression Inventory. ^a^ Data available for 98% of sample. ^b^ Data available for 81% of patients.

Participants from the BRCDECC study included patients with depression who had experienced two or more depressive episodes of at least moderate severity and met DSM-IV diagnostic criteria for recurrent major depressive disorder (APA, 1994), assessed using the Schedules for Clinical Assessment in Neuropsychiatry (Wing et al., 1990). The healthy controls were interviewed to ensure they had never experienced a depressive episode. Exclusion criteria included a diagnosis of a neurological disorder, head injury leading to loss of consciousness, or conditions known to affect brain structure or function (including alcohol or substance misuse), ascertained during clinical interview. Potential participants were also excluded if they or a first-degree relative had ever fulfilled criteria for mania, hypomania, schizophrenia or mood-incongruent psychosis. Current depressive symptoms were measured using the Beck Depression Inventory (BDI) (Beck et al., 1961).

Participants from the VIBES study included euthymic patients with bipolar disorder and healthy individuals. The diagnostic status of all participants was assessed using the Structured Clinical Interview for DSM-IV for Axis I diagnoses (First et al., 2002a; First et al., 2002b). Patients fulfilled criteria for bipolar disorder type I according to the Diagnostic and Statistical Manual of Mental Disorders, 4th edition, revised (DSM-IV) (APA, 1994). Healthy controls were selected based on the absence of family history and personal lifetime history of psychiatric disorders. To ensure that patients were in remission, their psychopathology was assessed weekly over a period of 1 month prior to testing and at each assessment they scored below 7 in the Hamilton Depression Rating Scale (HDRS) (Hamilton, 1960) and the Young Mania Rating Scale (YMRS) (Young et al., 1978). Patients were also required to have remained on the same type and dose of medication for a minimum of 6 months. All participants were free of any medical comorbidity and had no lifetime history of substance dependence or substance abuse in the six months leading to their brain scan.

The BRCDECC study was approved by the Bexley and Greenwich Research Ethics Committee, and the VIBES study was approved by the Ethics Committee of the Institute of Psychiatry and the South London and Maudsley National Health Service Trust. Written informed consent was obtained from all participants.

### 2.2 DNA extraction and genotyping

DNA was obtained from all participants using buccal swabs and/or blood. Participants from the VIBES study were genotyped on the Psych Chip (Illumina Infinium PsychArray-24) and participants from the BRCDECC study were genotyped using the Illumina HumanHap610-Quad BeadChip. SNP positions were lifted over from hg18 to hg19 build using the UCSC LiftOver tool. The data were imputed using the Michigan Imputation Server (Das et al., 2016) (https://imputationserver.sph.umich.edu/index.html) with 1000 Genomes as reference set utilizing SHAPEIT and Minimac software. Data quality was controlled in PLINK v1.07 (Purcell et al., 2007). In short, SNPs were excluded when missingness >1%, minor allele frequency (MAF) <1%, or Hardy-Weinberg equilibrium (HWE) *p* < .00001, and participants were excluded when missingness >1%. Sex and relatedness checks were carried out, in addition to principal component analyses, to confirm self-reported ethnicities (Patterson et al., 2006).

### 2.3 Polygenic risk scores

Genome-wide polygenic risk scores (PRSs) for ADHD, Autism Spectrum Disorder, Bipolar Disorder, and Schizophrenia were generated with PRSice software (http://prsice.info/) (Euesden et al., 2014), using the most recent Psychiatric Genomics Consortium genome-wide association analyses within the cross disorder study (Cross-Disorder Group of the Psychiatric Genomics Consortium, 2013) and schizophrenia GWAS (Ripke et al., 2014), available from the PGC website (www.med.unc.edu/pgc/results-and-downloads). The SNPs used were *P*-value-informed clumping in PLINK with a cut-off of r^2^ = 0.25 within a 200-kb window, and excluding the MHC region of the genome because of its complex linkage disequilibrium structure. For each participant, PRSs were generated using SNPs with a *p*-value threshold of <0.1 in the panel of SNPs from the Psychiatric Genomics Consortium (see Table S1 in the supplement for discovery sample sizes from the Psychiatric Genomics Consortium, and the number of SNPs included for each PRS). None of the individuals in our study were included in the GWAS data used to identify the SNPs. Since we calculated 5 psychiatric PRS scores, we decided to use the P-threshold of <0.1 as it has been shown that progressive P-thresholds at liberal thresholds explain more variance in the clinical phenotype (Ruderfer et al., 2014).

### 2.4 MRI data acquisition

For participants from the BRCDECC study, T1-weighted structural images were acquired using a 1.5-Tesla General Electric Signa MR Imaging system (General Electric, Milwaukee, WI, USA), in the sagittal plane (magnetization-prepared rapid gradient-echo sequence; repetition time (TR) = 8.592 ms, echo time (TE) = 3.8 ms, inversion time (TI) = 1000 ms, slice thickness = 1.2 mm, voxel dimensions = 0.939 × 0.937 × 1.2 mm, matrix size 192 × 192, field of view = 240, flip angle = 8°) (Cole et al., 2013).

For participants from the VIBES study, T1-weighted structural images were acquired using a 1.5-Tesla GE Neuro-optimised Signa MR system (General Electric, Milwaukee, WI, USA), in the axial plane (inversion recovery prepared, spoiled gradient-echo sequence; TR = 18 ms, TE = 5.1 ms, TI = 450 ms, slice thickness = 1.5 mm, voxel dimensions = 0.9375 × 0.9375 × 1.5 mm, matrix size 256 × 192, field of view = 240, flip angle = 20°, number of excitations = 1) (Dima et al., 2016a; Frangou et al., 2017).

### 2.5 MRI data processing

All MRI images were pre-processed using a standard voxel-based morphometry (VBM) pipeline (Ashburner and Friston, 2000), implemented in Statistical Parametric Mapping (SPM12) (www.fil.ion.ucl.ac.uk/spm/software/spm12/), running on Matlab 2016 (Math Works, USA).

The origins of all images were manually set to the anterior commissure. The images were then segmented into grey matter, white matter, and cerebrospinal fluid using unified segmentation. The Diffeomorphic Anatomical Registration using Exponential Lie algebra (DARTEL) algorithm (Ashburner, 2007) was applied to the segmented brain tissues to generate a study-specific template, and images were normalised to the template using non-linear warping.

Normalization to standard space, including smoothing using an 8 mm full-width-half-maximum Gaussian kernel, was done in two ways; including a “modulation” step or not. Modulation scales the grey matter probability values after spatial normalization to ensure that the total amount of grey matter in each voxel is conserved after warping (Mechelli et al., 2005). This is recommended for univariate voxel-wise analysis of grey matter volumes. However, for a multivariate analysis, when the *relationship* between grey matter intensities across voxels is modelled, it might be better to preserve concentrations of grey matter (i.e. not to modulate images) because modulation could alter the inter-relationship between voxel values. Hence, we use modulated images for the univariate analysis, and non-modulated images for the multivariate analysis. Total intracranial volume (including grey matter, white matter and cerebrospinal fluid) was calculated for all individuals for inclusion in the analyses as covariates.

To explore potential differences in grey matter volume between patients and controls, we used a standard univariate voxel-based morphometry (VBM) analysis in SPM12 (Ashburner and Friston, 2000), in the two studies separately. The input features were the smoothed, modulated, normalised grey matter images. Covariates (included as nuisance regressors) were age, sex, and intracranial volume. An explicit grey matter mask was applied. Two-sample *t*-tests were conducted (for the two studies separately). The significance threshold was set using family-wise error (FWE) correction (*p* < .05), and we report clusters larger than k = 20.

### 2.6 Univariate regression analysis

We first investigated the relationship between grey matter volume and polygenic risk scores (for ADHD, autism, bipolar disorder, and schizophrenia) in our sample of 213 individuals, using a standard, univariate voxel-based morphometry (VBM) analysis in SPM12 (Ashburner and Friston, 2000).

The input features for the univariate multiple regression were the smoothed, modulated, normalised grey matter images. Covariates (included as nuisance regressors) were age, sex, intracranial volume, status (patient, control), and study (BRCDECC, VIBES). An explicit grey matter mask was applied. We looked for regions with either a linear increase or decrease in grey matter volume associated with higher PRS (i.e. both positive and negative associations). We report clusters remaining significant after family-wise error (FWE) correction (*p* < .05, with a cluster forming threshold of *p* < .001), and regions surviving FWE correction at the voxel level (p < .05).

### 2.7 Multivariate regression analysis

We then tested whether a multivariate pattern of grey matter predicts polygenic risk scores for the five psychiatric disorders (ADHD, autism, bipolar disorder, and schizophrenia) in the whole sample including 213 individuals. This was done using multivariate Relevance Vector Regression (RVR) (Tipping, 2001) implemented in the Pattern Recognition for Neuroimaging Toolbox (PRoNTo; Schrouff et al., 2013) (www.mlnl.cs.ucl.ac.uk/pronto) running on Matlab 2016.

RVR is a probabilistic kernel-based pattern recognition method using Bayesian inference to obtain sparse regression models, and allows the extraction of patterns within a high-dimensional feature space (such as voxel-based intensities representing local grey matter concentration). This method has been described previously (Araque Caballero et al., 2016; Gong et al., 2014; Moradi et al., 2017; Tognin et al., 2014).

The input features for the multivariate pattern analyses were the smoothed, normalised grey matter images. Covariates – including age, sex, group status (patient, control), study (BRCDECC, VIBES), and intracranial volume – were regressed out from the training data, and the same transformation was applied to the test data within the cross-validation framework. The RVR was trained using a leave-one-subject-out cross-validation. This is a frequently used validation method, involving leaving one participant out for test and train the model on N-1 participants, and doing so N times so that each participant is left out once. We also repeated our analyses leaving 10% of the sample out (i.e. a ten-fold cross-validation). Because our sample size was not divisible by ten, 9 folds contained 21 individuals and 1-fold contained 24 individuals. These additional results are presented in the supplement (Table S6).

We report the Pearson correlation coefficient and the Mean Squared Error (MSE; normalised to the range of the predicted variable) between the actual and predicted PRS. These reflect how well a multivariate pattern of grey matter predicts an individual's PRS; a correlation of 1 would indicate that the predicted PRS is identical to the actual PRS, and an MSE of 0 would mean that there is no error in this prediction. Note that in a multivariate analysis results do not imply whether there is a positive or negative association between grey matter and PRS, instead it is the pattern of grey matter across the brain – which could include both increases and decreases – that predicts the PRS.

The significance of both the correlation coefficient and the MSE were estimated using a permutation test whereby the target data were randomised and the model re-run 1000 times (with the lowest *p*-value attainable being 1/1000 = 0.001). We also present regions (from the Automatic Anatomical Labelling (AAL) atlas), and their weights, that contribute the most to estimations of PRSs. Importantly, however, in a multivariate analysis all voxels contribute to the predictions and we present the regional contributions for visualization purposes only. As described in Schrouff et al. (2013; pg. 232), “this is because it is the combination of all weights that defines the model, and the weights at each voxel are dependent of one another and no direct lozalization or voxel-wise statistical test assuming independence can be performed on them”.

Since we are conducting four multivariate analyses (for the five different polygenic risk scores), we correct *p*-values for multiple testing using false discovery rates (FDR) implemented in Matlab using the Benjamini and Hochberg (1995) procedure. We decided to use the FDR correction by Benjamini and Hochberg since the four PRS scores are not independent.

As secondary analyses, to investigate whether associations are driven by subgroups of participants, we repeated the multivariate regression models including only patients (*N* = 102), only patients with major depression (*N* = 69) or bipolar disorder (*N* = 33), as well as including only healthy controls (*N* = 111).

## 3 Results

Participants' characteristics are presented in Table 1.

### 3.1 Polygenic risk scores

Patients with depression, bipolar disorder and healthy controls did not differ in mean ADHD, autism, or schizophrenia PRS. However, the three groups differed in the bipolar disorder PRS (F(2,210) = 12.05, *p* = 1.11 × 10^−5^), with patients with bipolar disorder having the highest risk, followed by patients with major depression, and lastly controls. See Table 2. Raw scores across the three groups are presented in the supplement (Table S2, Fig. S1).

**Table 2:** **Polygenic risk scores (PRS) for the four disorders.** Shown are tests for overall group differences (F tests), and the differences from controls in standardised z scores (to controls' means and standard deviations, SD) for patients with depression and patients with bipolar disorder (± SD).

**Polygenic Risk Score (PRS)**	**F test, p-value**	**Patients with Major Depression (vs. HC)**	**Patients with Bipolar Disorder (vs. HC)**	**p-values for two-sample t-tests**^**⁎**^
ADHD PRS	F(2,210) = 0.18, *p* = .83	0.09 ± 0.92	0.09 ± 1.13	NS
Autism PRS	F(2,210) = 1.68, *p* = .19	0.04 ± 1.05	0.36 ± 0.88	NS
Bipolar Disorder PRS	F(2,210) = 12.05, p = 1.1 × 10^−5^	0.40 ± 0.46	0.77 ± 0.95	HC vs MDD patients *p* = .002 HC vs BPD patients *p* = 8.6 × 10^−6^ MDD vs BPD patients *p* = .043
Schizophrenia PRS	F(2,210) = 0.77, *p* = .47	0.12 ± 0.42	0.06 ± 0.59	NS

*Comparison between groups (two-sample t-tests, uncorrected for multiple testing), for polygenic risk scores with significant overall group differences (i.e. Bipolar Disorder PRS).

NS = Not significant

ADHD = Attention Deficit Hyperactivity Disorder; BPD = Bipolar Disorder; HC = Healthy Controls; MDD = Major Depressive disorder.

Note: The control group includes controls from both samples (*N* = 111)

Pairwise correlations showed that the schizophrenia PRS was significantly associated with scores for ADHD (*r* = 0.20, p_FDR_ = 0.01) and bipolar disorder (*r* = 0.60, p_FDR_ = 2.2 × 10^−15^), and the ADHD and bipolar risk scores were also correlated (*r* = 0.23, p_FDR_ = 0.0007). The other pairwise correlations were not significant. See the supplement for full results (Table S3, Fig. S2).

### 3.2 Univariate analysis results

We explored differences in grey matter volume between patients and controls using VBM analysis separately in the depression BRCDECC and the bipolar VIBES study. No significant results were found.

We investigated the association between grey matter volumes and PRSs using univariate multiple regression, in the whole sample of 213 individuals. No results remained significant after family-wise error (FWE) correction for ADHD, autism, or bipolar disorder, PRS.

For the schizophrenia PRS, no cluster remained significant after FWE cluster correction, however one region (including 24 voxels, peak MNI coordinates: x = 46, y = −78, z = 28 mm, right inferior occipital gyrus) remained significant at the voxel-level, with a FWE-corrected peak-level *p*-value of 0.005 (z-value = 4.99); increased PRS was associated with increased grey matter volumes.

Un-thresholded t-maps for associations with the four PRSs are shown in the supplement (Fig. S3).

### 3.3 Multivariate regression results

**Fig. 1:**
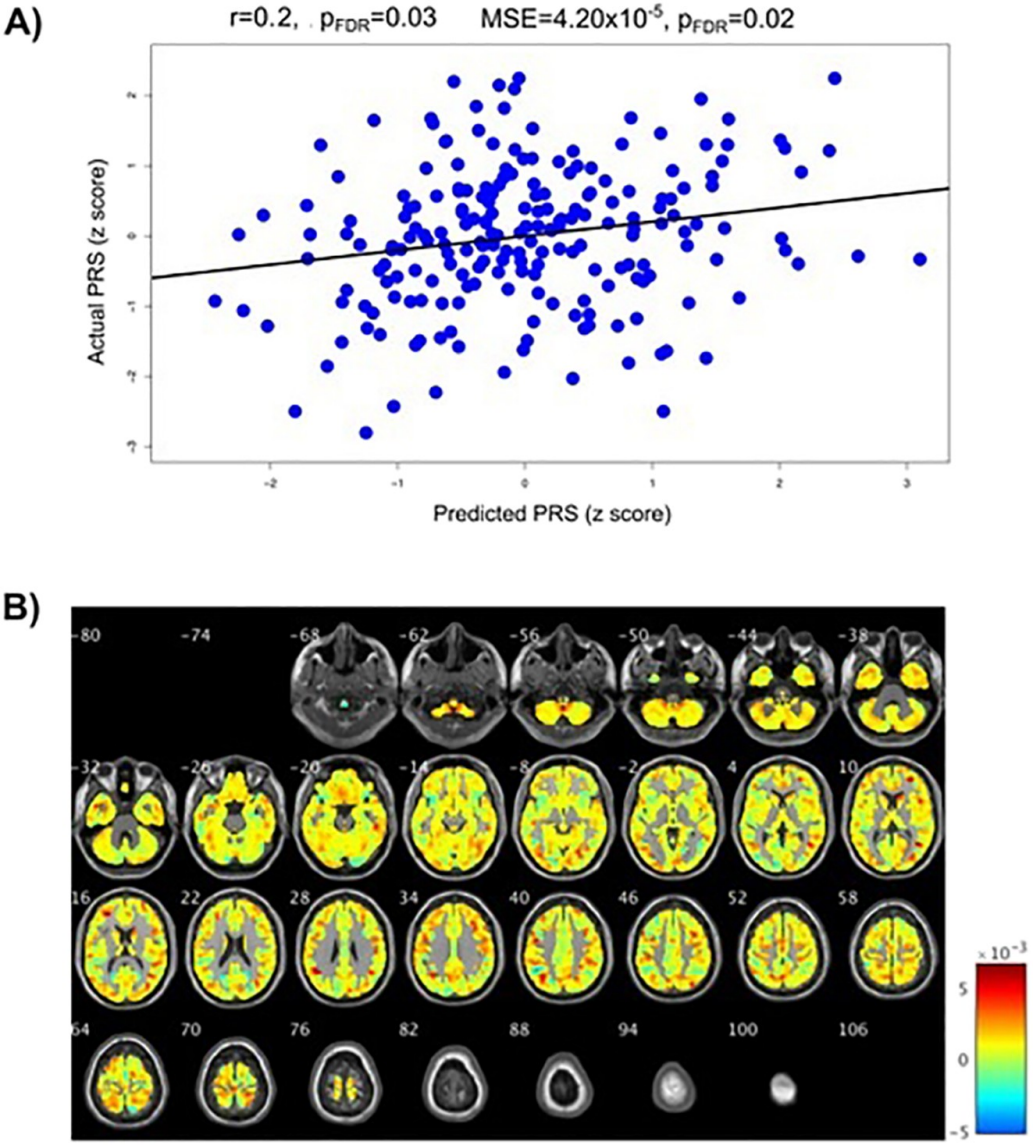
**Results for the autism polygenic risk score (PRS). A)** Correlations between actual autism PRS and the PRS predicted from the grey matter volume maps. p_FDR_ = False Discover Rate corrected *p*-values. **B)** Weight maps of contribution of voxels across the whole brain for the predicted autism PRS shown in A (in the whole sample, *N* = 213).

**Fig. 2:**
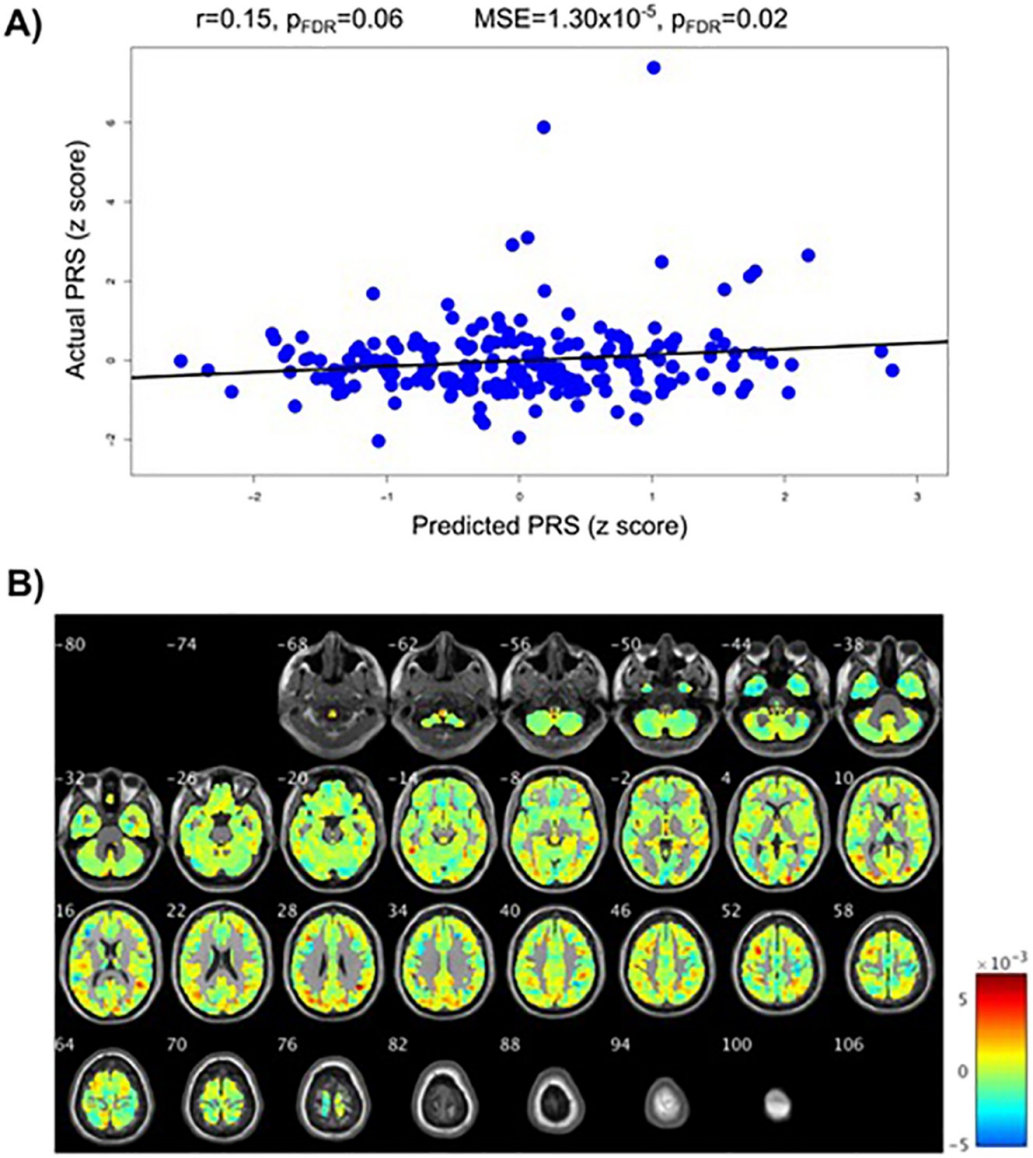
**Results for the schizophrenia polygenic risk score (PRS). A)** Correlations between actual schizophrenia PRS and the PRS predicted from grey matter volume maps. p_FDR_ = False Discover Rate corrected p-values. **B)** Weight maps of contribution of voxels across the whole brain for the predicted schizophrenia PRS shown in A (in the whole sample, N = 213).

We then investigated the association between brain structure and PRSs using multivariate relevance vector regression with the leave-one-subject-out cross-validation. In the whole sample of patients and healthy controls, a multivariate pattern of grey matter intensity significantly predicted the PRS for autism (*r* = 0.20, p_FDR_ = 0.03, MSE = 4.20 × 10^−5^, p_FDR_ = 0.02). Associations with the schizophrenia PRS were significant when looking at the mean squared error (MSE = 1.30 × 10^−5^, p_FDR_ = 0.02), and the correlation (*r* = 0.15, p_unc_ = 0.032); however the correlation did not survive FDR correction (p_FDR_ = 0.06). The PRSs for ADHD and bipolar disorder were not significantly associated with grey matter across the whole sample. These results are presented in Table 3 and Figs. 1A and 2A.

**Table 3:** **Multivariate pattern recognition results.** Correlations (r) between the actual and predicted polygenic risk scores (PRS) – from grey matter volumes – and (normalised) mean squared errors (MSE) in the whole sample (N = 213).

**ADHD PRS**	**Autism PRS**	**Bipolar Disorder PRS**	**Schizophrenia PRS**
*r* = −0.11	r = 0.20	*r* = −0.01	*r* = 0.15
p_unc_ = 0.821 p_FDR_ = 0.821	p_unc_ = 0.008 p_FDR_ = 0.032	p_unc_ = 0.518 p_FDR_ = 0.690	p_unc_ = 0.032 p_FDR_ = 0.064
MSE = 6.19 × 10^−5^	MSE = 4.20 × 10^−5^	MSE = 5.18 × 10^−5^	MSE = 1.30 × 10^−5^
p_unc_ = 0.950 p_FDR_ = 0.950	p_unc_ = 0.005 p_FDR_ = 0.020	p_unc_ = 0.266 p_FDR_ = 0.354	p_unc_ = 0.012 p_FDR_ = 0.024

ADHD = Attention Deficit Hyperactivity Disorder; p_unc_ = uncorrected p-value; p_FDR_ = False Discovery Rate corrected p-value.

Importantly, the results were stable across folds as illustrated by the proximity of the expected ranking to the actual rankings for the regions with the five highest weights (see Table S4 and Fig. S4 in the supplement). The expected ranking indicates how consistent the ranking is across cross-validation folds; if the expected ranking is close to the actual ranking of a region (by weight) then the result is considered stable across the folds. For the autism PRS the left inferior parietal gyrus contributed 1.60% to the regression and the expected ranking was 1.00. For the left angular gyrus these values were 1.43% and 2.12, the left occipital lobe 1.38% and 3.15, the left cuneus 1.32%, 4.18 and left precentral gyrus 1.30%, 5.10. For the schizophrenia PRS the right angular gyrus contributed 1.53% to the regression and the expected ranking was 1.11. For the right superior occipital lobe these values were 1.48% and 2.40, the cerebellar vermis 1.05% and 3.09, left middle occipital lobe 1.34%, 4.37 and left superior occipital lobe 1.33%, 4.83. While these weights are in keeping with the proportion of brain volume for each region, they are low, indicating that the pattern of associations is widespread across the brain; this is seen in Figs. 1B and 2B where the weight maps of contribution of voxels across the whole brain to prediction of PRSs are shown.

As secondary analyses, to investigate whether associations are driven by subgroups of participants, we repeated the multivariate regression models including only patients (*N* = 102), only patients with major depression (*N* = 69) or bipolar disorder (*N* = 33), as well as including only healthy controls (*N* = 111). None of these analyses were significant and these results are presented in the supplement (Table S5). We also repeated the analysis using a randomly selected subsample of 100 individuals (comprising both patients and controls), in order to investigate whether this lack of prediction above was likely due to the reduced sample size. This analysis was also non-significant (Table S7).

We also re-ran our analyses using a 10-fold cross-validation. This resulted in findings very similar to the original analyses, and are presented in Table S6 in the supplement.

## 4 Discussion

We aimed to investigate associations between polygenic risk scores (PRSs) for four psychiatric disorders (ADHD, autism, bipolar disorder and schizophrenia) and brain volumes. Results showed that the PRS for autism and schizophrenia were associated with a multivariate pattern of grey matter concentrations in this sample of healthy controls and psychiatric patients. PRSs for ADHD and bipolar disorder, however, could not be predicted by brain structure in this study. These findings lend support to the hypothesis that the polygenic liability for autism and potentially schizophrenia is associated with changes in grey matter concentrations across the brain.

Both autism and schizophrenia are complex neurodevelopmental disorders that are highly polygenic. Our findings show that the PRSs for these two disorders are not correlated, indicating that although both reflect commonly occurring genetic risk factors, these loci are not shared between these disorders. This is consistent with previous studies finding low genetic correlations between these two disorders in terms of common genetic variation (Cross-Disorder Group of the Psychiatric Genomics Consortium, 2013; Lee et al., 2013). For autism, although many rare variants such as de novo mutations and copy number variants have been identified (Buxbaum, 2009; Ronemus et al., 2014; Sanders et al., 2015), no single nucleotide polymorphism (SNP) has yet been significantly detected (Warrier et al., 2015). That we find a significant association with brain structure is in line with arguments that the PRS captures causal variation not yet significantly identified in genome-wide association studies (Wray et al., 2014).

For schizophrenia, over 100 common SNPs have been detected (Ripke et al., 2014). The discovery sample used to calculate the PRS for schizophrenia is substantially larger than for any of the other disorders investigated here, making the schizophrenia PRS relatively powerful (Purcell et al., 2009; Ripke et al., 2014). We know that larger discovery sample sizes will lead to more true positive variants being included in the risk score, and hence the score being a more sensitive marker of genetic risk (Dudbridge, 2013; Wray et al., 2014). Once discovery samples sizes increase for the other disorders and more significant loci are identified, we might expect to see more significant associations in the kind of analyses conducted in this study, if there is a true link between psychiatric genetic risk and brain structure.

Given the lack of SNPs associated with autism and the smaller discovery sample size compared to schizophrenia for the PRS calculation, it is interesting that we find associations between brain structure and the autism PRS. If replicated, this might be due to the early development of autism and its association with severe lifelong neurodevelopmental symptoms (Baxter et al., 2015). Hence, associations between genetic risk for autism and brain structure might be relatively large compared to disorders that emerge later in life. Furthermore, Lee et al. (2013) found that the proportion of variance explained by SNPs located in genes that are expressed in the central nervous system is relatively high for autism compared to other psychiatric disorders investigated here. It could thus be suggested that genes associated with autism influence brain structure to a relatively large degree and is more widespread than first suspected.

We see these associations between PRS for autism and schizophrenia and grey matter in a sample not including patients affected by these disorders, indicating that this is not driven by the expression of the disease, but by the genetic risk captured by the PRSs. Furthermore, there was no evidence in our sample that the associations between PRS and grey matter patterns were moderated by diagnostic status (MDD or bipolar), and this indicates that disease status or use of medication is not driving the associations. However, we acknowledge that MDD and bipolar disorder patients were a subset of the overall sample.

We found no evidence for associations between brain grey matter and genetic risk for bipolar disorder, or ADHD. When it comes to ADHD, since this disorder has been found to be less heritable with larger environmental influences (Brikell et al., 2015; 2015; Franke et al., 2011;), the lack of associations here are perhaps unsurprising. However, given the large genetic overlap between bipolar disorder and schizophrenia, seen both in the correlation between these scores in this study and in the literature (Craddock et al., 2005; Cross-Disorder Group of the Psychiatric Genomics Consortium, 2013; Lee et al., 2013; Lichtenstein et al., 2009), it is somewhat surprising that we observed a trend level association for schizophrenia but not for bipolar disorder. This might be due to the significantly larger discovery sample size used to calculate the risk score for schizophrenia. Furthermore, compared to autism, bipolar disorder develops later in life, which could explain these lack of findings; maybe genetic risk associated with these disorders do not have as strong an influence on structural neurodevelopment as that for autism.

In a multivariate analysis, all voxels contribute to predictions and it is not possible to single out whether any region is predictive in isolation. In our study, maybe the most important conclusion from the weight maps is that the pattern of grey matter predicting PRS is widespread across the whole brain; large regions identified (voxel size ~5000) and very small weights attributed even to the top regions with weights of no >1.6%. This widespread multivariate pattern justifies the use of multivariate machine learning models, since it will likely be more difficult to identify significantly associated regions using a univariate method (Cohen et al., 2017; Woo et al., 2017). This is evident in our findings, especially for the autism PRS where we find a multivariate association but no significant univariate results. Nevertheless, an important goal of future work is to use multivariate methods that might be able to provide more insight into what brain regions are most important for predictions, such as sparse network-based methods or elastic net classifiers. Another feature of the multivariate analysis is that the output is a weight map which does not speak to whether these areas have increased or decreased grey matter volumes in relation to PRS scores. The univariate analysis for the PRS for schizophrenia revealed only one significant positive correlation between schizophrenia PRS and the right inferior occipital gyrus, contributing to the argument of using multivariate machine learning methods.

We consider this to be a proof-of-principle study, and while our sample size is relatively large for imaging research, it is small when compared to genetic studies. Furthermore, although we use cross-validation (including the leave-out-out method in the main paper, as well as a ten-fold cross-validation in the Supplement), these findings need replication in large independent samples to be confirmed, ideally in a large sample of healthy individuals not constrained by disease status. A limitation of the PRS is that because it is an estimate of the combined genetic risk carried by an individual, it does not tell us what specific variants are most important for the association observed. Therefore, future research should investigate whether a subset of common genetic variants (e.g. on a functional pathway) are more strongly influencing grey matter structure. These findings identify brain correlates of PRS, but do not inform on the functional role of these systems in conferring risk, which is an important question as more data becomes available, including developmental and longitudinal data. Further limitations of this study include the use of MRI data from two different scanners, which might have influenced results (although this was included as a covariate in all analyses), and that we did not have both neuroimaging and genetic data available from patients with autism, ADHD, or schizophrenia. Lastly, the method applied in this paper does not allow to test for PRS by diagnosis interaction.

In summary, this was the first study using a multivariate approach – both for genetic and imaging data – to investigate associations between brain grey matter and polygenic risk scores for four psychiatric illnesses. We show that both the autism and the schizophrenia risk scores are significantly predicted by grey matter structure. These results support the hypothesis that cumulative genetic risk for autism and schizophrenia is associated with changes in grey matter concentrations across the brain. Autism develops early in life and is associated with severe neurodevelopmental symptoms, including brain structural changes. This is likely contributing to our current findings, suggesting genetic risk for autism might be associated with relatively large changes in brain structure even in individuals not expressing the illness. That the pattern of associations was widespread across the brain, as expected, supports the use of a multivariate approach to detect these patterns of changes in grey matter.

## Appendix A Supplementary data

Supplementary material Image 1
