# Supplementary material for: Associations between polygenic risk scores for four psychiatric illnesses and brain structure using multivariate pattern recognition
Source: Neuroimage Clin. 2018 Oct 9;20:1026–36. doi: 10.1016/j.nicl.2018.10.008 (PMC6197704; doi:10.1016/j.nicl.2018.10.008)
Supplement: Supplementary file 1 — Supplementary material [file mmc1.docx]

**Supplemental Material**

**Table S1.** Discovery sample sizes, from the Psychiatric Genomics Consortium (Cross-Disorder Group of the Psychiatric Genomics Consortium, 2013; Ripke *et al.*, 2014), and the number of single nucleotide polymorphisms (SNPs) included for each polygenic risk score (PRS) at the p-value threshold (p_T_) of p < 0.1.

| **Polygenic risk score (PRS)** | **Discovery sample size** | **SNPs included**  **(p_T_ < 0.1)** |
| --- | --- | --- |
| Attention Deficit Hyperactivity Disorder (ADHD) PRS | 1,947 trio cases; 1,947 trio pseudocontrols;  840 cases; 688 controls | 13,372 |
| Autism Spectrum Disorder PRS | 4,788 trio cases; 4,788 trio pseudocontrols;  161 cases; 526 controls | 14,161 |
| Bipolar Disorder PRS | 6,990 cases; 4,820 controls | 14,367 |
| Schizophrenia PRS | 36,989 cases; 113,078 controls | 37,894 |

**Table S2.** Polygenic Risk Scores (PRS) for the four disorders across the three participant groups.

|  | **Healthy Controls** (N=111) | | **Patients with Major Depression** (N=69) | | **Patients with Bipolar Disorder** (N=33) | |
| --- | --- | --- | --- | --- | --- | --- |
|  | mean | SD | mean | SD | mean | SD |
| **ADHD PRS** | 9.7x10^-4^ | 3.1x10^-4^ | 1.00x10^-3^ | 2.8x10^-4^ | 1.00x10^-3^ | 3.5x10^-4^ |
| **Autism Spectrum Disorder PRS** | -1.62x10^-3^ | 2.1x10^-4^ | -1.61x10^-3^ | 2.2x10^-4^ | -1.54x10^-3^ | 1.9x10^-4^ |
| **Bipolar Disorder PRS** | 6.27x10^-3^ | 4.7x10^-4^ | 6.46x10^-3^ | 2.1x10^-4^ | 6.63x10^-4^ | 4.4x10^-4^ |
| **Schizophrenia PRS** | -2.49x10^-3^ | 1.5x10^-4^ | -2.51x10^-3^ | 6x10^-5^ | -2.48x10^-3^ | 9x10^-5^ |
| ADHD = Attention Deficit Hyperactivity Disorder; SD = Standard Deviation | | | | | | |

**Figure S1. Violin plots showing Polygenic Risk Scores (z scores) across clinical groups.** ADHD = Attention Deficit Hyperactivity Disorder, BPD = Patients with Bipolar Disorder, CNT = Controls, MDD = Patients with major Depressive Disorder


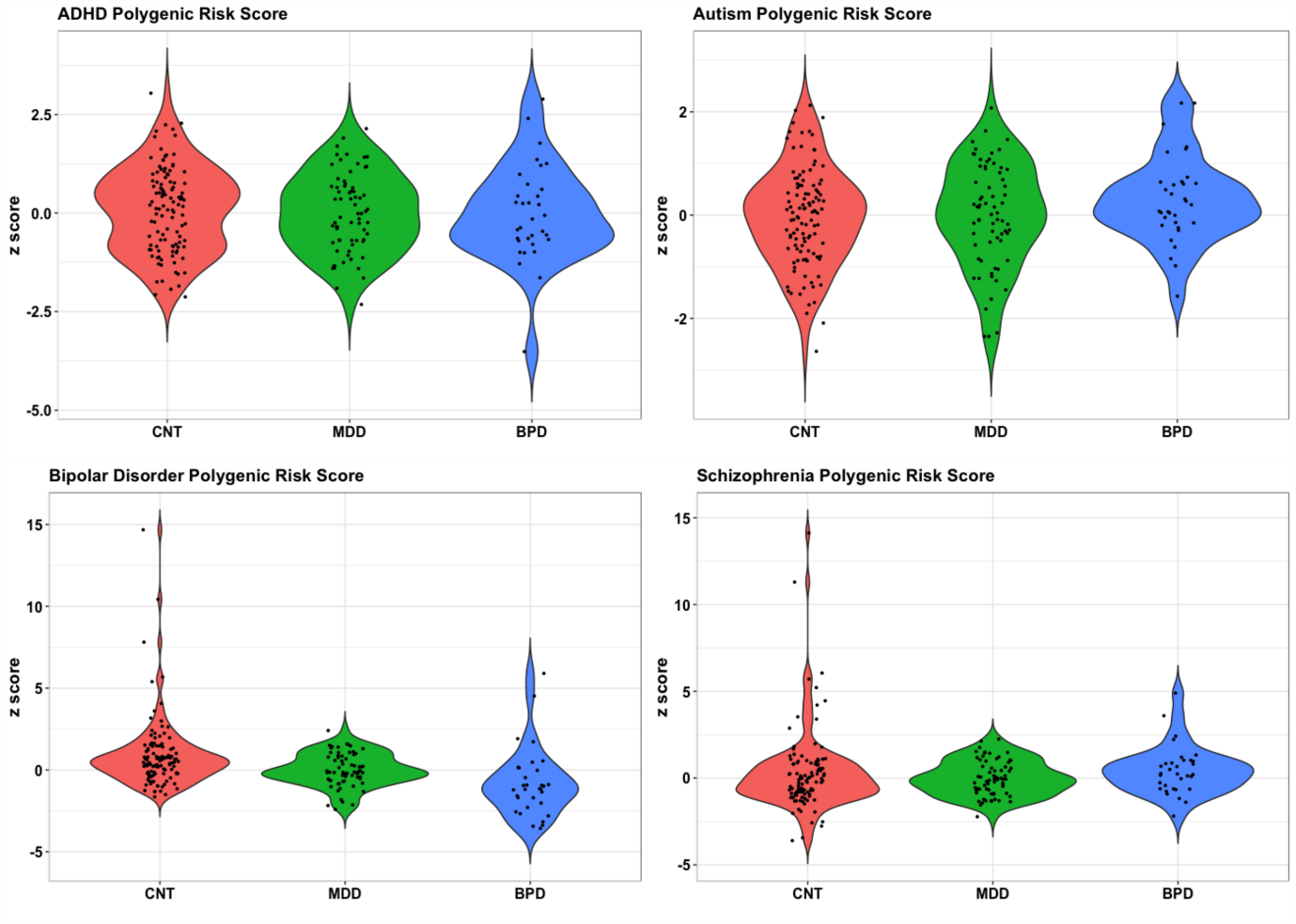


**Table S3.** Correlations between the four polygenic risk scores (PRS). Shown are pairwise Pearson’s correlations (r) and p-values.

|  | **ADHD PRS** | **Autism Spectrum Disorder PRS** | **Bipolar Disorder PRS** |
| --- | --- | --- | --- |
| **Autism Spectrum Disorder PRS** | r = -0.001,  p_unc_ = 0.992  p_FDR_ = 0.992 |  |  |
| **Bipolar Disorder PRS** | *r = 0.230,*  *p_unc_ = 0.001*  *p_FDR_ = 0.004* | r = 0.054,  p_unc_ = 0.437  p_FDR_ = 0.624 |  |
| **Schizophrenia PRS** | *r = 0.201,*  *p_unc_ = 0.003*  *p_FDR_ = 0.010* | r = 0.064,  p_unc_ = 0.355  p_FDR_ = 0.592 | *r = 0.604,*  *p_unc_ = 2.2x10^-16^*  *p_FDR_ = 2.2x10^-15^* |

**Figure S2.** A scatterplot between the Schizophrenia PRS and the Bipolar Disorder PRS. We also performed a Spearman's correlation between the Schizophrenia and Bipolar Disorder PRS. The results showed that the correlation remained significant: rs = 0.139, p-value = 0.043.


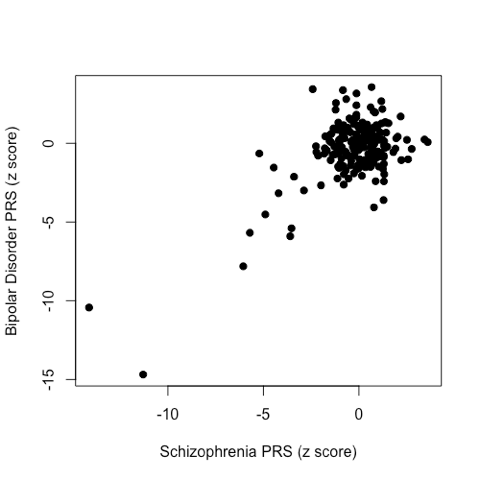


**Figure S3.** Un-thresholded t-maps of univariate regression results across the four Polygenic Risk Scores (PRS). ADHD = Attention Deficit Hyperactivity Disorder.


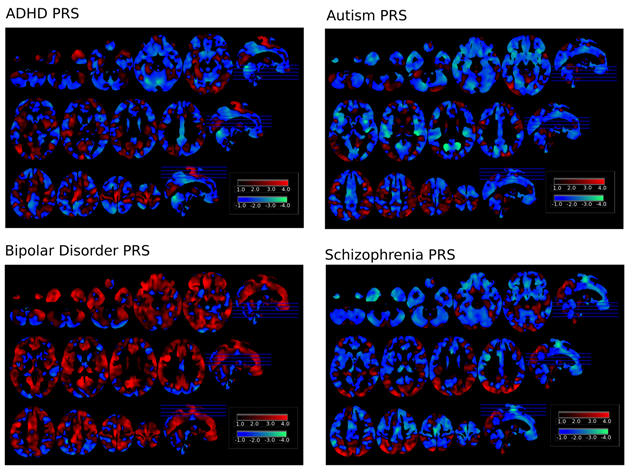


**Figure S4.** Weight maps for brain areas contributing the most to predictions of (a) autism and (b) schizophrenia polygenic risk scores. Brain areas were identified using the Automatic Anatomical Labelling (AAL) atlas. Note that all voxels contribute to the predictions and we show the top regions only for visualization purposes.
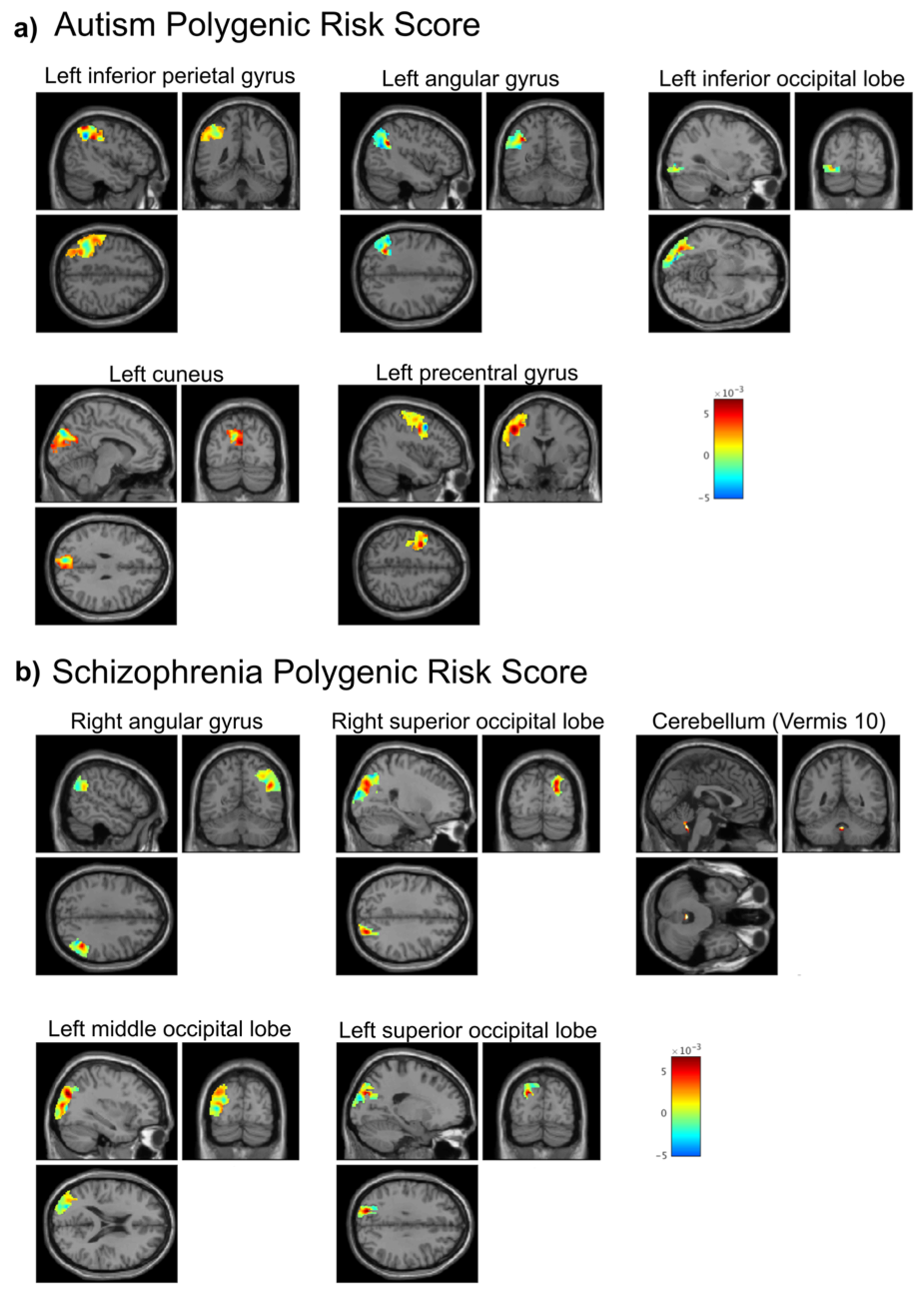


**Table S4. The top 5 most predictive regions for the two significant models in the whole sample (N=213) showing the close proximity of expected to actual ranking.** Brain areas were identified using the Automatic Anatomical Labelling (AAL) atlas. Shown is the contribution (weight, in %) and the size (in voxels) of the regions, as well as the expected ranking. The expected ranking indicates how consistent the ranking is across cross-validation folds; if the expected ranking is close to the actual ranking of a region (by weight) then the result is considered stable across the folds. Note that all voxels contribute to the predictions in a multivariate analysis, in the tables and figures we present only the top regions.

| ***Autism Polygenic Risk Score*** | | | |
| --- | --- | --- | --- |
| **Region (in order of weight ranking)** | **Weight** | **Size (voxels)** | **Expected ranking** |
| Left inferior parietal gyrus | 1.60% | 5052 | 1.00 |
| Left angular gyrus | 1.43% | 2481 | 2.08 |
| Left inferior occipital lobe | 1.38% | 1979 | 3.15 |
| Left cuneus | 1.32% | 3029 | 4.18 |
| Left precentral gyrus | 1.30% | 5523 | 5.10 |
| ***Schizophrenia Polygenic Risk Score*** | | | |
| **Region (in order of weight ranking)** | **Weight** | **Size (voxels)** | **Expected ranking** |
| Right angular gyrus | 1.53% | 3364 | 1.11 |
| Right superior occipital lobe | 1.48% | 2439 | 2.40 |
| Cerebellum (Vermis 10) | 1.45% | 40 | 3.09 |
| Left middle occipital lobe | 1.34% | 6784 | 4.37 |
| Left superior occipital lobe | 1.33% | 2200 | 4.83 |

**Table S5.** Multivariate Relevance Vector regression results in subgroups of participants; correlations (r) between the actual and predicted polygenic risk scores and (normalised) mean squared errors (MSE). P-values uncorrected for multiple testing.

| **Polygenic Risk Score (PRS)** | **All patients**  **(N=102)** | **Patients with Bipolar Disorder**  **(N=33)** | **Patients with Depression**  **(N=69)** | **Healthy**  **controls**  **(N=111)** |
| --- | --- | --- | --- | --- |
| ADHD PRS | r=-0.22, p=0.903,  MSE=6.61x10^-5^, p=0.970 | r=-0.61, p=0.984  MSE=9.92x10^-5^, p=0.993 | r=-0.15, p=0.694,  MSE=7.89x10^-5^, p=0.900 | r=-0.06, p=0.594,  MSE=7.77x10^-5^, p=0.839 |
| Autism PRS | r=0.08, p=0.209  MSE=4.71x10^-5^, p=0.153 | r=0.13, p=0.155  MSE=4.14x10^-5^, p=0.155 | r=-0.01, p=0.420  MSE=5.40x10^-5^, p=0.317 | r=0.06, p=0.240, MSE=4.57x10^-5^, p=0.266 |
| Bipolar Disorder PRS | r=-0.16, p=0.824  MSE=5.67x10^-5^, p=0.508 | r=-0.46, p=0.954  MSE=1.26x10^-4^, p=0.870 | r=-0.33, p=0.930  MSE=5.53x10^-5^, p=0.862 | r=0.0001, p=0.410, MSE=7.17x10^-5^, p=0.386 |
| Schizophrenia PRS | r=0.01, p=0.392  MSE=1.34x10^-5^, p=0.533 | r=0.12, p=0.190  MSE=1.77x10^-5^, p=0.229 | r=-0.08, p=0.541  MSE=1.59x10^-5^, p=0.560 | r=0.10, p=0.151, MSE=2.06x10^-5^, p=0.088 |
| ADHD = Attention Deficit Hyperactivity Disorder | | | | |

**Table S6. Multivariate Relevance Vector regression results using a ten-fold cross-validation method, i.e. leaving 10% of the sample out.** Correlations (r) between the actual and predicted polygenic risk scores (PRS) – from grey matter volumes – and (normalised) mean squared errors (MSE) in the whole sample (N=213).

| **ADHD PRS** | **Autism PRS** | **Bipolar Disorder PRS** | **Schizophrenia PRS** |
| --- | --- | --- | --- |
| r = -0.07 | r = 0.21 | r = -0.10 | r = 0.16 |
| p_unc_ = 0.717  p_FDR_ = 0.794 | p_unc_ = 0.014  p_FDR_ = 0.056 | p_unc_ = 0.794  p_FDR_ = 0.794 | p_unc_ = 0.083  p_FDR_ = 0.166 |
| MSE = 6.07x10^-5^ | MSE = 4.17x10^-5^ | MSE = 5.46x10^-5^ | MSE = 1.37x10^-5^ |
| p_unc_ = 0.910  p_FDR_ = 0.910 | p_unc_ = 0.010  p_FDR_ = 0.038 | p_unc_ = 0.485  p_FDR_ = 0.646 | p_unc_ = 0.019  p_FDR_ = 0.038 |

**Table S7.** **Multivariate Relevance Vector regression results using a randomly selected subsample of 100 individuals.** Correlations (r) between the actual and predicted polygenic risk scores (PRS) – from grey matter volumes – and (normalised) mean squared errors (MSE). P-values uncorrected for multiple testing.

| **ADHD PRS** | **Autism PRS** | **Bipolar Disorder PRS** | **Schizophrenia PRS** |
| --- | --- | --- | --- |
| r = -0.086  p_unc_ = 0.610  p_FDR_ = 0.610 | r = -0.002  p_unc_ = 0.422  p_FDR_ = 0.562 | r = 0.114  p_unc_ = 0.129  p_FDR_ = 0.258 | r = 0.196  p_unc_ = 0.137  p_FDR_ = 0.148 |
| MSE = 6.780x10^-5^  p_unc_ = 0.846  p_FDR_ =  0.846 | MSE = 4.492x10^-5^  p_unc_ = 0.544  p_FDR_ = 0.726 | MSE = 6.904x10^-5^  p_unc_ = 0.122  p_FDR_ = 0.244 | MSE = 1.582x10^-5^  p_unc_ = 0.034  p_FDR_ = 0.136 |
